# A phase III wait-listed randomised controlled trial of novel targeted inter-professional clinical education intervention to improve cancer patients’ reported pain outcomes (The Cancer Pain Assessment (CPAS) Trial): study protocol

**DOI:** 10.1186/s13063-018-3152-z

**Published:** 2019-01-18

**Authors:** Jane L. Phillips, Nicole Heneka, Melanie Lovell, Lawrence Lam, Patricia Davidson, Frances Boyle, Nikki McCaffrey, Sally Fielding, Tim Shaw

**Affiliations:** 1 University of Technology Sydney, PO Box 123, Ultimo, NSW 2007 Australia; 2 University of Sydney, City Rd, Camperdown, NSW 2006 Australia; 3 Johns Hopkins University, 3400 N. Charles Street, Baltimore, MD 21218 USA; 4 Deakin University, 1 Gheringhap St, Geelong, VIC 3220 Australia

**Keywords:** MeSH terms, Cancer pain, Pain measurement, Assessment, pain, Health services research, Patient-reported outcome measures, Palliative care, Clinical competence, Education, professional, Mobile applications, Cost-benefit analysis

## Abstract

**Background:**

Variations in care models contribute to cancer pain being under-recognised and under-treated in half of all patients with cancer. International and national cancer pain management guidelines are achievable with minimal investment but require practice changes. While much of the cancer pain research over the preceding decades has focused on management interventions, little attention has been given to achieving better adherence to recommended cancer pain guideline screening and assessment practices. This trial aims to reduce unrelieved cancer pain by improving cancer and palliative doctors’ and nurses’ (‘clinicians’) pain assessment capabilities through a targeted inter-professional clinical education intervention delivered to participants’ mobile devices (‘mHealth’).

**Methods:**

A wait-listed, randomised control trial design. Cancer and/or palliative care physicians and nurses employed at one of the six participating sites across Australia will be eligible to participate in this trial and, on enrolment, will be allocated to the active or wait-listed arm. Participants allocated to the active arm will be invited to complete the mHealth cancer pain assessment intervention. In this trial, mHealth is defined as medical or public health practice supported by mobile devices (i.e. phones, patient monitoring devices, personal digital assistants and other wireless devices). This mHealth intervention integrates three evidence-based elements, namely: the COM-B theoretical framework; spaced learning pedagogy; and audit and feedback. This intervention will be delivered via the QStream online platform to participants’ mobile devices over four weeks. The trial will determine if a tailored mHealth intervention, targeting clinicians’ cancer pain assessment capabilities, is effective in reducing self-reported cancer pain scores, as measured by a Numerical Rating Scale (NRS).

**Discussion:**

If this mHealth intervention is found to be effective, in addition to improving cancer pain assessment practices, it will provide a readily transferable evidence-based framework that could readily be applied to other evidence practice gaps and a scalable intervention that could be administered simultaneously to multiple clinicians across diverse geographical locations. Moreover, if found to be cost-effective, it will help transform clinical continuing professional development. In summary, this mHealth intervention will provide health services with an opportunity to offer an evidence-based, pedagogically robust, cost-effective, scalable training alternative.

**Trial registration:**

Australian New Zealand Clinical Trials Registry (ANZCTR), ACTRN12618001103257. Registered on 3 July 2018.

**Electronic supplementary material:**

The online version of this article (10.1186/s13063-018-3152-z) contains supplementary material, which is available to authorized users.

## Background

Effective pain management cuts across professional boundaries with clinicians’ failure to routinely screen and assess for pain contributing to the burden of unrelieved cancer pain experienced by 39–66% of cancer patients [1]. To date, most research has focused on testing new cancer pain treatments, with little attention payed to strengthening cancer pain screening and assessment practices. Evidence suggests that implementing routine screening and assessment and managing pain in accordance with evidence-based guidelines can improve quality of care and outcomes for cancer pain [2–4].

Evidence of screening and assessment practices are increasingly being recognised as quality indicators of optimal cancer pain management internationally, with pain now recognised as the fifth vital sign and routinely recorded on inpatients’ observation charts [5]. The subjective nature of pain makes measuring patient-reported outcomes the optimal source of clinical information [6]. However, instead of routinely seeking a patient-reported Numerical Rating Scale (NRS) pain score, many clinicians adopt informal pain screening approaches [7] and/or fail to comprehensively assess their patients’ pain [8, 9]. A survey of Australian patients receiving community palliative care found that one-third experienced moderate to severe pain, restricting their activities over the preceding three days [10]. When this survey was repeated with a different cohort, a similar burden of unrelieved pain was reported [11].

It is recommended that a comprehensive pain assessment be undertaken for all cancer patients with an NRS pain score ≥ 2 [12, 13]. Determining the location, temporal pattern(s), exacerbating and/or relieving factors associated with the patient’s pain and ascertaining whether the pain has nociceptive or neuropathic origin(s) [14] is integral to determining a differential pain diagnosis and an individualised cancer pain management plan. Undertaking a comprehensive pain assessment is complex and too few clinicians have been formally taught how to assess across these pain domains, with most learning ‘on the run’, by observing their peers or through industry events. Yet, clinicians’ cancer pain assessment competencies [15], their understanding of the most suitable pain assessment tools, commitment and capacity to integrate pain assessment findings into clinical decision-making [16], communication skills and capacity to address their patients’ care needs within the context of multi-professional practice [17], all impact on their patients’ pain outcomes.

Unfortunately, many interventions aimed at improving clinicians’ pain assessment have had a limited effect on cancer pain outcomes [18–25]. The reasons for this failure are complex and varied and include failure to address the multiple barriers at the patient (i.e. failing to report the presence of pain), clinician (i.e. failing to adhere to recommended routine pain screening and assessment practices) [18] and organisational/system (i.e. accreditation and funding not linked to meeting pain management standards) levels. Most previous clinician-targeted cancer pain management interventions have been based on intuition, as opposed to being theoretically driven [26], which in part explains their ad-hoc success and limited transferability [16]. While a small number of previous interventions have focused on inter-professional education [18–23], not all have optimised the educational intervention ‘dose’ (strength) [18–22] or effectively managed the complexity of the intervention [20] and/or been underpinned by an evidence-based behavioural change framework [26, 27].

Despite the complexity of undertaking a comprehensive pain assessment, very few, if any, interventions have targeted cancer pain assessment as a distinct learning component, with most embedding screening and assessment into a broader cancer pain management learning package, privileging pharmacological cancer pain management practices [8]. In addition, few previous clinician targeted cancer pain management interventions have engaged both doctors and nurses, with most focusing exclusively on educating a single discipline as opposed to inter-professional practices [19]. Yet, the interdisciplinary nature of cancer and palliative care necessitates the implementation of targeted inter-professional learning strategies addressing pain assessment as a stand-alone construct [19]. Given this reality, there are opportunities to maximise the impact of any clinically focused cancer pain assessment behavioural change intervention by including evidence-based strategies, such as: (1) audit and feedback, which includes any summary of clinical performance over a defined period and can lead to potentially important improvements in practice [28]; (2) comprehensive interventions, which are more effective when addressing patients’ pain, especially when documentation and monitoring interventions are combined [18]; (3) more intense interventions involving extensive follow-up, a comprehensive educational program and greater resource allocation which are significantly more likely to impact positively on reducing cancer pain [20]; and (4) explicit application of theory to identify contextual conditions necessary for their success and enhance learning [26]. In addition, given the significant annual investments healthcare organisations make towards building clinical capabilities, few continuing professional development (CPD) activities are underpinned by evidence-based pedagogy and/or have been subjected to an economic analysis.

These gaps highlight the need to develop and test alternative strategies with a real potential to build cancer and palliative care clinicians’ cancer pain assessment capabilities, using effective and cost-effective pedagogically sound interventions underpinned by evidence-based behavioural change theories [27].

The trial protocol reported here adheres to the Standard Protocol Items: Recommendations for Interventional Trials (SPIRIT) checklist [29].

## Objectives

### Primary objective

To determine if a tailored mHealth intervention targeting clinicians’ cancer pain assessment capabilities is effective in reducing self-reported cancer pain scores, as measured by a NRS.

### Secondary objectives

To determine if the intervention:

i. increases clinicians’ adherence to the Australian Cancer Pain Management in Adults Guidelines’ screening and assessment recommendations;
ii. increases the quality of clinicians’ pain assessment documentation;
iii. increases clinicians’ cancer pain assessment capabilities (knowledge and confidence); and
iv. is cost-effective compared with standard cancer and palliative clinician CPD activities at reducing cancer patients’ reported pain scores from a healthcare systems perspective.

## Trial design

**Fig. 1:**
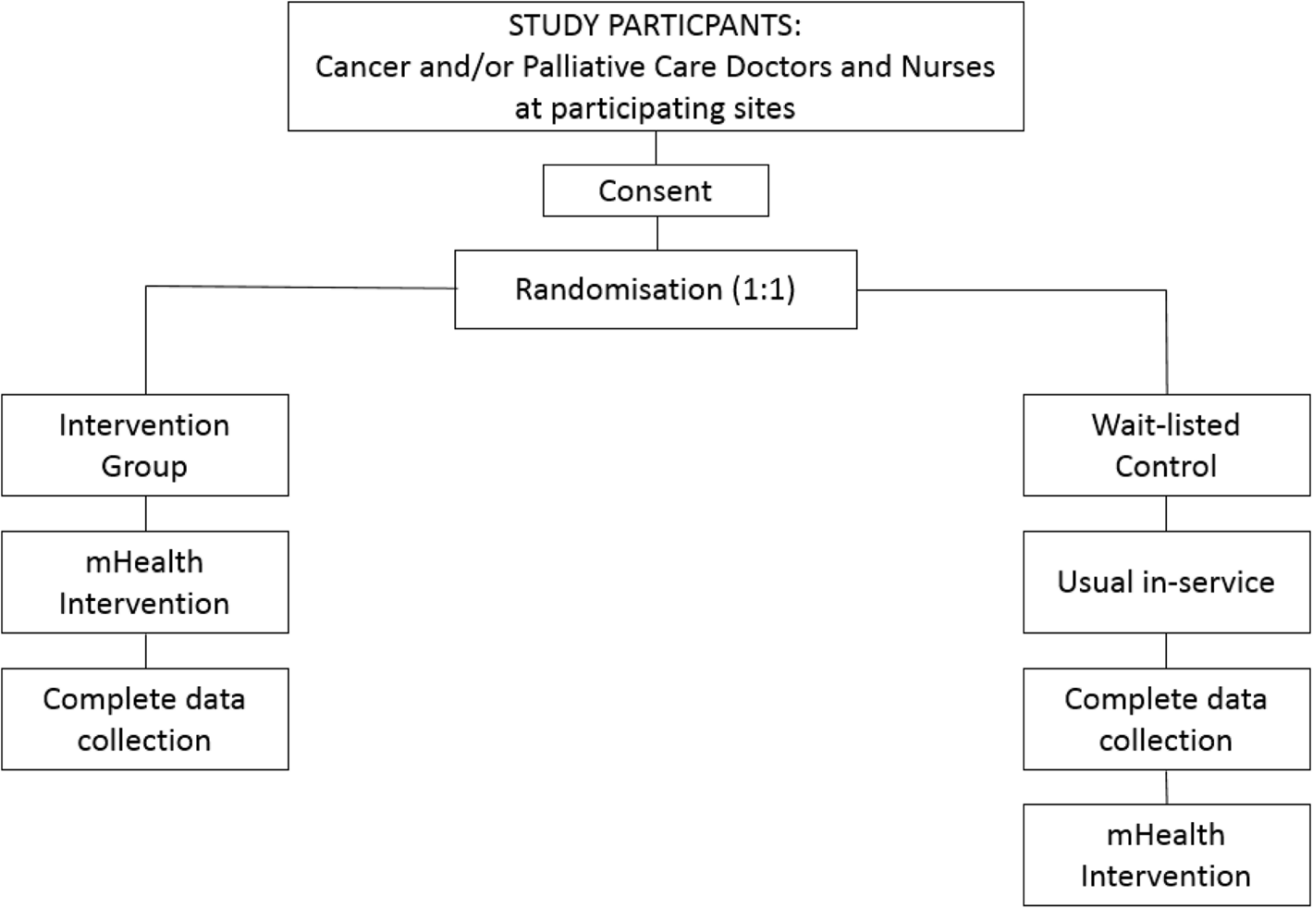
Study *flow chart*

The study will use a phase III wait-listed randomised controlled trial (RCT) design, with individual cancer and palliative care clinicians as the units of randomisation (Fig. 1).

### Design considerations

The design of the study is a simple RCT with all participants considered as the basic randomisation unit and not nested in different sites. The justification for such consideration was based on the following two observations made during the pilot study: (1) there was virtually no cross-contamination among all participants, in that they had no knowledge about each other’s study status; and (2) there were no clustering characterisation across different sites such that participants could be considered as from a random sample [30]. Based on these observations, the study team opted to adopt the simple RCT design instead of a cluster RCT design.

## Methods: participants, interventions and outcomes

### Study setting

Six palliative and/or cancer care centres in metropolitan New South Wales (NSW), Australia will be involved in the trial. Details about participating sites can be obtained from the trial web address: http://www.ANZCTR.org.au/ACTRN12618001103257.aspx.

### Eligibility criteria

#### Sites

All participating sites must nominate a contactable ‘clinical champion’ with whom the project team can liaise directly.

#### Participants

All medical and nursing personnel (‘clinicians’) routinely caring for cancer and/or palliative care patients at a participating site are eligible to participate in the study. Participants must be willing to give written informed consent and be willing to participate in and comply with the study policies and procedures.

### Exclusion criteria

#### Sites

A competing trial that is enrolling cancer and/or palliative care clinicians.

#### Participants

Agency and/or casual nurses or physicians who have worked less than one shift in the month before the intervention commences and unregistered health professionals who are unlikely to be undertaking and documenting patients’ pain assessment.

### Intervention

#### Intervention to be tested

A clinician-focused, spaced learning pain assessment performance feedback intervention (‘intervention’) combining: (1) an online spaced learning module delivering authentic case-based cancer pain assessment scenarios directly to participants’ mobile devices; (2) real-time site-specific pain assessment audit and feedback, providing de-identified peer-to-peer comparisons; and (3) online links to evidence-based pain assessment decision supports.

#### Development of the intervention

**Fig. 2:**
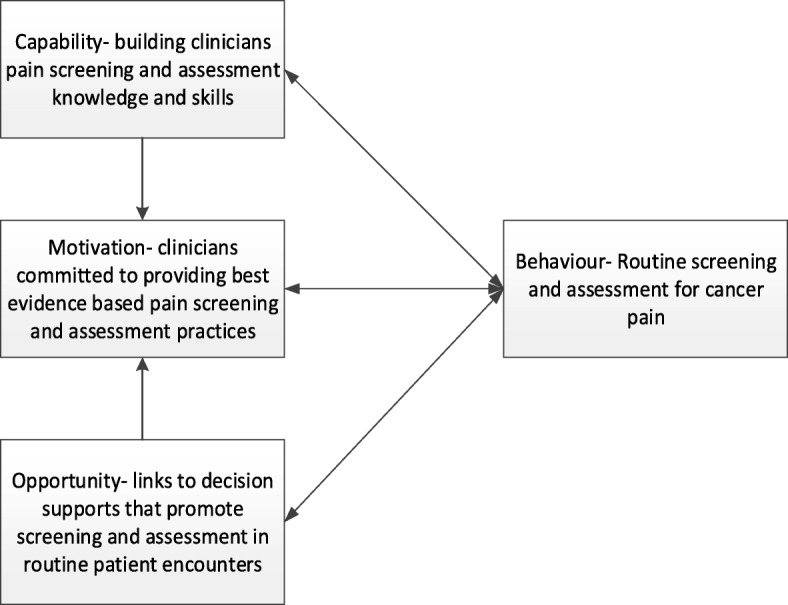
Applying the COM-B system to the CPAS trial

The research team has undertaken a program of work [30, 31], underpinned by the Medical Research Council’s (MRC) Framework [32] for complex interventions, to better understand and address the cancer pain assessment evidence practice gap. The development phase involved an environmental scan, review of cancer patient reported pain ratings, audit of cancer pain assessment practices and development of a tailored mHealth intervention designed to increase cancer and palliative care clinicians’ capacity to effectively screen and comprehensively assess their patients’ cancer pain. This mHealth intervention integrates several evidence-based elements, namely: (1) spaced learning; (2) audit and feedback and use of a clinical champion; and (3) a theoretical framework (COM-B System) (Fig. 2).

#### Spaced learning

Learning encounters which are ‘spaced’ and ‘repeated over time’ (‘spaced learning’) result in more efficient learning and improved retention compared to the traditional face-to-face bolus distribution learning format [33, 34]. A recent systematic review has identified that when delivered prospectively, spaced learning generates significant topic-specific knowledge [35]. Spaced learning differs significantly from other pedagogies in that it ‘pushes’ short, clinically focused, case-based scenarios to a participant’s email, which take ≤ 5 min to answer every other day. Upon answering a question, a participant’s de-identified performance is compared to that of their peers and they are provided with succinct feedback and links to relevant evidence-based resources and decision supports. The spaced learning cancer pain assessment case-based learning scenarios were developed by an expert panel comprising palliative and cancer care clinicians as well as education specialists, using the CASE methodology, a systematic framework for generating evidence-based, authentic case scenarios for learning [36]. These case-based learning scenarios have been tested in our pilot and feasibility studies [30, 31].

#### Audit and feedback

There is moderate evidence that auditing professional practice and feeding back the results encourages greater adherence to professional standards and/or guidelines [28]. A recent RCT demonstrated the value of integrating actual performance data into interventions designed to change clinicians’ behaviour in a simulated environment [37]. The quality and safety data feedback, as part of a spaced learning intervention, impacted positively on US surgical residents’ knowledge retention and simulated central line insertion performance [37]. While there is ample RCT evidence that spaced learning can improve knowledge acquisition and increase knowledge retention from three months and up to two years [33, 34, 37]), there are no robust data that spaced learning can impact positively on clinical practices outside of a simulated environment [35]. In our intervention, real-time site-specific pain assessment audit and feedback, providing de-identified peer-to-peer comparisons will be integrated into the spaced learning case scenarios. As there is good evidence that audit and feedback is most effective when provided by a supervisor or colleague (‘clinical champion’) [28], the photo and a statement from the site-specific clinical champion will also be integrated into the case scenario feedback script. Participants will be provided with hyperlinks to evidence-based pain assessment decision supports.

#### Theoretical framework

The intervention is underpinned by the COM-B System (Fig. 2), a framework for understanding and targeting behaviour change [27]. In this framework, capability, opportunity and motivations interact to generate the desired clinical behaviour. In this study, clinicians’ capabilities include having the necessary knowledge and skills to routinely screen for pain and to proceed to complete a comprehensive pain assessment if the patient reports a NRS pain ≥ 3. Motivation energises and directs clinician behaviour including conscious decision-making about routine screening and assessing to make cancer pain screening and assessment possible. Opportunity includes all factors that lie outside of the individual that prompt clinicians to make cancer pain screening and assessment possible (access to online decision prompts and strategies for integrating pain assessment into usual care). Adopting the evidence-based spaced learning pedagogy adds strength to this theoretically driven behaviour-change intervention. The Qstream™ platform will be used to deliver the spaced learning case-based cancer pain assessment scenarios and relevant site-specific pain assessment performance data.

#### Pilot testing

The piloting phase tested the acceptability of the spaced learning intervention [30] and the feasibility of the proposed RCT [31]. Our uncontrolled pilot demonstrated a significant reduction in the mean patient-reported average daily numerically rated pain scores (0–10), between the pre-post test audit, of 1.5 (95% confidence interval [CI] = 0.7–2.3) [30]. In our pre-post test feasibility study, a positive effect was observed in all measures in the study. A larger adequately powered RCT is required to confirm these results given these previous studies had insufficient power to demonstrate statistical significance. These pilot study results have informed the proposed RCT sample size [30, 31]. This manuscript focuses on the protocol for the evaluation phase, which aims to determine if a tailored online spaced learning intervention that increases clinicians’ cancer pain assessment capabilities translates into a clinically significant reduction in cancer and palliative care patients’ NRS pain scores.

#### Intervention arm

**Fig. 3:**
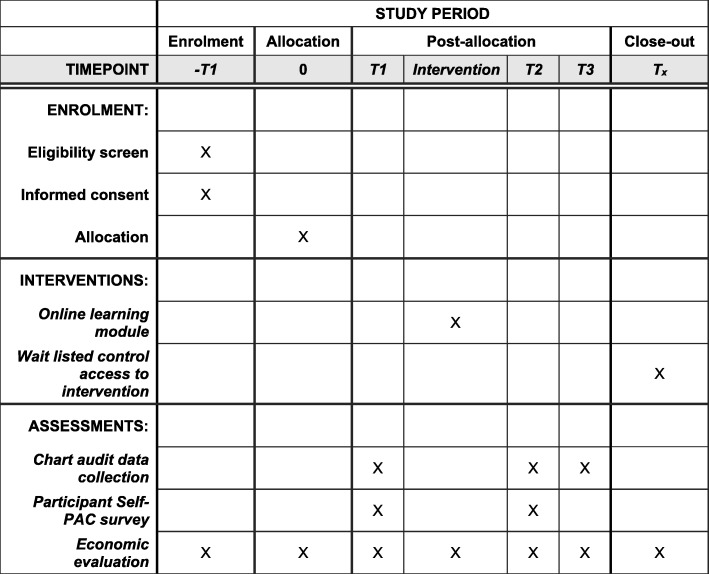
Standard Protocol Items Recommendations for Interventional Trials (SPIRIT) figure [5] showing the schedule of enrolment, interventions and assessments for the Cancer Pain Assessment Study Trial

Consenting participants randomised to the intervention arm will receive the intervention immediately (Fig. 3). During the intervention, the participants will receive four real-time site-specific pain assessment audit and feedback data-related questions as well as eight case-based cancer pain screening and assessment scenarios (‘case studies’). The intervention will be delivered via Qstream™, the online spaced learning platform. Each case will be delivered directly to participant’s mobile devices (via a free app) or email. Participants will receive two case studies every second day, with each case taking approximately 3–5 min to answer. Upon answering a case, participants will receive: immediate de-identified feedback on how they have performed compared to their peers; and/or succinct audit and feedback data regarding their sites pain screening or assessment practices, with site-specific messages from local clinical champions. They will also receive links to relevant evidence-based resources and the Australian Cancer Pain Management in Adults Guidelines decision support [12]. Correctly answered cases will be re-sent after eight days, incorrectly answered cases will be re-sent every five days. Cases will no longer be sent once they have been correctly answered twice. While the study period for each participant will vary depending on how long it takes them to complete the intervention, it is estimated that it will take no longer than four weeks, as detailed in Fig. 3.

#### Control group

Consenting participants randomised to the wait-listed control group will complete all of the data collection measures but will not have access to the intervention until the intervention arm participants at each site have completed the trial and all data collection has been completed. It is anticipated that the control group participants will have access to the intervention within 16 weeks of the trial commencing at each site.

#### Adherence to intervention

Intervention adherence will be monitored weekly via reporting analytics built into the QStream™ platform. All participants will receive notification via their nominated email and/or app, when a new case study is available to answer, and/or if there are case studies awaiting their completion. Weekly engagement reports will be generated via the Qstream™ platform to monitor participants’ progress. Participants who are not progressing through the intervention as planned will be emailed directly and offered online or face-to-face technical support, as required. Participants’ individual progress will remain confidential and will not be disclosed to the participating service; however, de-identified, weekly progress reports will be sent to the each site’s nominated ‘clinical champion’ to identify the percentage of participants who have enrolled and completed the intervention. The research team will be available to assist participants with any technical aspects of the intervention, such as app download, and help troubleshoot any other technical issues that may arise.

### Outcomes

#### Primary outcome measure

The primary outcome measure will be at the patient level and concern the probability that a tailored mHealth performance feedback intervention will translate to a clinically meaningful reduction in the mean change in the pain NRS (0–10) scores, from admission to census date. A cut-off of a two-point difference or 30% reduction in pain intensity on a NRS is considered to represent a clinically important difference [38].

As this is a pragmatic trial, all pain NRS scores will be captured during the chart audit process from the patients’ medical records, thus negating the need for patient consent. The NRS is the optimal brief measure of pain severity on the basis of compliance rates, responsiveness, ease of use and applicability; it is also recommended by the Australian Cancer Pain Management in Adults Guidelines [12]. The inter-rater reliability of the chart auditors will be calculated using: Kappa statistics for categorical variables; and the Bland–Altman method of plots and limits of agreement for continuous variables will be used to determine the degree of agreement between chart auditors [39]. A waiver of patient consent has been authorised by the Human Research Ethics Committee (HREC) to include documented de-identified pain screening and assessment data from all patients receiving care from the participating units/sites during the a priori defined audit periods at Times 1–3, to avoid selection bias.

#### Secondary outcome measures

A range of secondary outcomes will be examined, including clinicians’:

i. pain screening/assessment adherence scores;
ii. comprehensive pain assessment quality documentation scores; and
iii. Self-Perceived Pain Assessment Capabilities (Self-PAC) survey [40].

An economic evaluation will also be undertaken and will include the following outcomes: efficacy measured using the NRS pain scores, adherence scores and Self-PAC scores; resource use, including the intervention, i.e. clinician and administration time and Qstream platform; and standard CPD, including clinician time.

### Participant timeline

See Fig. 3 for the participant timeline.

### Sample size

Informed by our pilot work [30, 31] and based on a standard RCT design [41], it is estimated that a sample size of 35 participants in each arm is required. This assumes a 5% significance level and 90% power of the study with an effect size of a reduction of mean patient-reported pain rating of 1.5 (+ 2.0). Allowing for an attrition rate of 25%, and 5% for possible inclusion of covariates in the analyses, will increase the RCT sample size to 90 clinicians (45 in each arm). To achieve the trial objectives, it is estimated that 90 cancer and/or palliative care clinicians, with half (*n* = 45) in each arm is required.

### Recruitment

Each participating site/unit will be invited to nominate a ‘clinical champion’ to act as the primary point of contact with the research team. Clinical champions will provide information about the study (Additional file 1) to all eligible participants within their department and encourage attendance at study information sessions conducted by the research team.

### Consent

All potential participants will be sent an email by the site clinical champion inviting them to attend an information session about the study, conducted by the research team. This email will include the Participant Information and Consent Form (Additional file 2) and information about the Qstream™ mobile application (Additional file 3). At the information session, participants will have the opportunity to discuss the study with a research team member(s) before consenting. Consent will be undertaken by a member of the research team. Upon consenting, participants will be asked to provide their work email address so a link to the baseline survey and Qstream™ enrolment instructions can be sent to them. Potential participants who are unable to attend the information session can contact the research team directly before consenting. The consent process for all participants at all sites will be the same.

### Allocation

Participants will be sequentially allocated a unique identifying number (ID) on consenting to be involved with the study. This ID number will consist of a two-digit site number; a sequential three-digit number will be allocated on randomisation of the participant. The full number sequence will be unique to that participant and will not be reassigned. This ID number will be used for all subsequent study documentation for that participant. Each participant will be allocated to an intervention or wait-listed control arm according to a randomisation schedule generated by a central registry at the study management centre (Bio-Statistician, LL). The central registry will be responsible for generating the randomisation tables and will provide the required number of randomisation sequences in a spreadsheet or word document. In order to avoid the difficulty of having imbalanced allocations, the permuted block randomisation method will be employed with a block size of 6–8 and a 1:1 ratio of intervention to control. The allocation of the randomisation codes will be managed by the study coordinator. The participant ID, allocation code, commencement and completion of the intervention surveys will be recorded in a log maintained by the study coordinator.

### Blinding

Study investigators are blinded to participant allocation; however, project officers collecting and managing the data will not be blinded, as the frequency of documented pain assessment between the intervention and control groups is a secondary outcome measure in this RCT.

### Methods: data collection, management and analysis

#### Data collection methods

Data will be collected at three time points during the trial—Time 1 (T1), Time 2 (T2) and Time 3 (T3) (Table 1)—as described below.

**Table 1:** Study activities at each time point

	Study time			
	Baseline (T1)	Intervention	T2	T3
	*Immediately before intervention commencing*		*Immediately following the completion of the intervention*	*12 weeks after completion of the intervention*
Study activities				
Chart audit data collection	X		X	X
Online Participant Survey (Self-PAC Survey)	X		X	
Economic data collection	X		X	X
Wait-listed control group access online module				X

##### Chart audit data

Patient-reported pain NRS scores and clinicians’ adherence to pain screening and assessment guidelines will be extracted from patients’ inpatient/ambulatory care medical records (electronic and/or paper-based). The chart audit tool developed, tested and refined during the pilot studies will be adopted to ensure standardised data collection. Medical records of all patients in the participating units, over 30 consecutive calendar days, will be screened for audit at each study time period (T1–T3). This will facilitate capturing chart audit data within the same duration across all participating units at each study time point. Documented patient-reported pain NRS scores and guideline adherence data will be collected by trained project officers.

Eligible medical records (chart audit) will include all patients who during the three audit periods: (1) have a primary diagnosis of cancer; 2) present for cancer treatment at a participating centre and/or are referred to a specialist cancer/palliative care service; and (3) have pain at the time of first visit/appointment/admission or develop pain during the audit period. In addition to capturing patients’ basic demographics such as age, gender, length of hospital stay, medical diagnosis, and cancer and pain treatment(s), the chart audit will also capture: patients’ pain scores on admission to the service; their pain scores at discharge and/or audit date, depending on which occurs first; clinicians’ documentation pain screening/assessment actions; and whether the clinician who documented the pain screening/assessment actions was an intervention participant or non-participant (T2 and T3 only).

Chart audit data collected at baseline (T1) will be integrated into the online learning intervention and tailored to each service (see ‘Intervention’ above). At T2 and T3, project officers will also record if pain screening/assessment actions documented in the patient chart were undertaken by an intervention participant or non-participant. This will be determined by matching clinician signatures in the patient chart with participant signatures on the study consent form and cross-referencing participants with the work roster for the audit period. To facilitate identification of participants versus non-participants, project officers undertaking the chart audits will not be blinded to the group allocation. Documentation of participant versus non-participant on the chart audit tool will be by way of a de-identified code only. No identifying participant details will be recorded on the chart audit tool.

##### Participant survey

The Self-PAC survey has undergone preliminary validation [40] and will be administered at two study time points (T1 and T2) in an online format. This instrument has three distinct sub-scales with Cronbach’s alpha reporting high internal consistency reliability: seven-item pain assessment knowledge (0.944); three-item pain assessment tool knowledge (0.846); and seven-item pain assessment confidence (0.919) scales [36]. To promote participant retention, project officers will send a unique survey link to all participants and send reminder emails to participants who do not complete the survey within the required time frame, at both survey time points.

##### Economic data

Efficacy (pain NRS score; adherence and Self-PAC scores) and resource use (intervention, i.e. clinician and administration time, Qstream™ platform; standard CPD, including clinician time) data will be collected. CPD activity data will be collected via survey and a pro-rata average cost per clinician calculated using the annual CPD site allocation.

#### Data management

All hard copies from the study will be kept at the Centre for Improving Palliative, Aged and Chronic Care through Clinical Research and Translation (IMPACCT), Faculty of Health, University of Technology Sydney, Level 3, 235 Jones Street, Ultimo, NSW 2007. The electronic list of study codes with participant details will be stored in the secure IMPACCT research drive and will be password-protected. Hard copies of data will be secured behind at least one locked door, within a locked filing cabinet. Electronic records will be protected by a password and the password will be changed at regular intervals. Data will be stored for seven years. A dedicated password protected REDCap account has been established to deliver participant surveys for this study. This account features enhanced security (SSL) and can only be accessed by authorised members of the Research Team. Survey data downloaded from the account will be password-protected and stored on a computer hard drive at IMPACCT in de-identified format. Once data has been downloaded and de-identified, the corresponding survey data will be deleted from the REDCap account.

#### Confidentiality

The participant will be enrolled into the study after the informed consent process has been completed. All consenting participants will be given a unique Participant Identification Code (ID). This will ensure that all identifying data (e.g. email address) can be removed before data analysis commences. This project ID will enable the research team to manage the data in a confidential manner. Participants are free to withdraw from the study once it has started and can do so at any time without having to give a reason. Withdrawal of consent will not affect participants’ employment at their current work site. Previously collected data may still be used in the analyses and participants will be advised it may not be possible to withdraw their data from the study results. Where possible, the reason for study withdrawal/non-completion will be collected.

#### Statistical methods

##### Statistical analysis plan

An intention-to-treat analysis will be applied to all primary and secondary outcomes. Missing data will be imputed using Multiple Imputation by Chained Equations. Should an imbalance in clinicians’ characteristics be found between groups at baseline, these characteristics will be included in the final analyses as covariates. A significance level of 5% will be adopted for refuting all test hypotheses. The intervention and wait-listed control arms will be compared on the primary and secondary outcome measures.

The primary endpoint (reduction in mean pain NRS scores) will be analysed using the Linear Mixed Method with a repeated measures approach and possible adjustments to patients’ and staff characteristics to compare the mean change in patient pain NRS scores from admission to discharge or audit date between the intervention and control groups across different time points.

The secondary endpoints will be analysed as follows:

i. The frequency of comprehensive pain assessment between the intervention and control will be determined by differences between groups. As the outcome variable is a count variable without a fixed bound, Poisson regression with possible adjustments for covariates will be applied to the data.
ii. A quality score will be calculated for each audited record across time and entered into the patient’s medical records. This score will reflect the quality of the pain documentation in the medical notes and will be calculated using seven items of documentation (pain severity score, location, radiation, aggravating and alleviating factors, quality and timing). One mark will be assigned to an item identified in the medical records and a summative quality score will then be calculated to represent the total amount of information recorded. A higher quality score represents a larger amount of pain assessment information recorded and a greater adherence to recommended pain assessment practices. The quality of pain assessment documentation will be determined by comparing the quality scores across time and between groups using a General Linear Model with repeated measures.
iii. The mean scores of the three domains of the Self-PAC survey (pain assessment knowledge, pain assessment tool knowledge and pain assessment confidence) will be compared across time and between the intervention and control groups using the General Linear Model with repeated measures approach and with possible adjustments for covariates effects.
iv. The primary objective of the cost-effectiveness analysis is to evaluate the incremental resource use, cost and consequences of adding the mHealth enabled pain assessment performance feedback intervention to standard clinician CPD activities to improve cancer pain control. A Markov decision model will be developed to estimate the cost-effectiveness of the mHealth intervention from a healthcare perspective. Healthcare resource utilisation and cost data will be estimated from a systematic literature review of the direct and indirect costs of pain in cancer patients including hospitalisations, emergency department visits, outpatient clinic appointments, medications, GP visits and investigations. Responder rates will be estimated from the project. The modelled economic evaluation will provide estimates of the incremental cost-effectiveness ratio (incremental cost per additional responder, response = a 2-point mean reduction in NRS pain score or 30% reduction in pain intensity) and the incremental net monetary benefit (INMB; monetary value of additional effects of care minus the additional costs of care) [42] at potential threshold values for responder rates and cost-effectiveness acceptability curves. Model sensitivity to variations in individual inputs and overall decision uncertainty will be assessed through probabilistic sensitivity analyses.

##### Interim analysis

An interim analysis will be undertaken immediately after 25 participants have completed the intervention with data collected at T2. At this time, the corresponding 25 clinicians in the control arm will also be assessed for T2 data collection as described in the protocol. The reasons for nominating a sample of 25 in both intervention and control arms are: (1) based on the concept that an interim analysis is recommendable at the halfway point of the trial (i.e. half of the RCT sample have completed the trial); (2) given that the total participants in each arm has been estimated to be 45, half of the sample will only be about 22 clinicians. This sample size would not be sufficient to support an accurate and precise comparison between groups. To ensure sufficient power for the interim analysis, a sample of 25 in each arm is considered reasonable. In order to stop the trial, we would need to demonstrate significant results of comparison between groups with an effect size of a reduction in mean patient-reported pain rating by at least 1.5 units in the intervention arm at an alpha level of 5% and preferably at 1%.

### Methods: monitoring

#### Data monitoring

As this trial does not directly involve patients, or include an intervention directed at patients, and there no adverse events anticipated as a result of the mHealth intervention, a data monitoring committee will not be convened. If any issues should arise, they will be dealt with at an extraordinary level through referral to an independent Trials Monitoring Committee.

#### Study monitoring/auditing

A study monitoring/auditing arrangement will be put in place to ensure that patient privacy has been protected, the study data can be verified and that the relevant approvals were in place. The arrangement will include: (1) regulatory approvals at each site are in place and filed according to protocol; (2) participating clinicians’ identity will be confirmed before study enrolment; (3) 10% of medical record audits at each site, at each time point, will be verified by a second auditor (project officer) to ensure consistent and accurate data collection as well as adequate protection of patient privacy; (4) project officers will record if pain screening/assessment actions documented in the patient chart were undertaken by an intervention participant or non-participant (T2 and T3 only), documentation of participant versus non-participant on the chart audit tool will be by way of a de-identified code only, i.e. no identifying participant details will be recorded on the chart audit tool; and (5) project officers to provide regular reports to the Investigator team on recruitment, data collection and any issues arising during these processes. Any perceived/identified irregularities will be referred to an independent Trials Monitoring Committee.

#### Harms

No harms are foreseen for this trial due to the nature of the intervention, which is an online learning module only and not a medical intervention. However, it is acknowledged that the intervention may evoke feelings of inadequacy or discomfort associated with previous experience caring for patients and/or their families with complex and/or poorly managed pain. The online module will promote reflective practice and adoption of best evidence-based pain assessment and management practices. The research team will be available if participants wish to explore their previous experiences and will refer participants onto appropriate support services, if required.

## Discussion

This trial will evaluate a targeted inter-professional mHealth clinical education intervention designed to drive innovation in cancer pain assessment. An adequately powered RCT is required to confirm our pilot results and to determine if an mHealth intervention that integrates spaced learning, audit and feedback, and decision support prompts can reduce cancer patients’ reported pain scores. A cost-effectiveness analysis is also required to determine whether the intervention represents value for money and should be promoted more widely as a cost-effective evidence-based intervention. Our proposed multicentred approach will increase the strength of the study recommendations and extrapolation of this intervention to other clinical settings.

If our phase III trial reports effectiveness, in addition to improving cancer pain assessment practices, it will provide an evidence-based framework (output) that could readily be used to address other entrenched evidence practice gaps (i.e. handwashing, managing the deteriorating patient and opioid prescribing). This intervention is also scalable as its mHealth delivery format ensures that the intervention can be readily rolled out across multiple services in diverse geographical locations. In the longer term, if the results are positive, they will help transform the CPD paradigm from one focused on random personal interests to one where clinicians’ learning experiences are aligned with identified clinical practice gaps, ensuring that future CPD policy supports learning experiences that improve patient care outcomes. While mHealth initiatives are increasingly being used, there is little research on which technologies or pedagogical approaches are most effective in promoting clinical practice behavioural change. There is even less evidence around the cost-effectiveness of these CPD interventions, hence the importance and timeliness of this study. Given the scarcity of health resources, any intervention designed to build clinicians’ pain assessment capacity needs to be cost-effective [43]. Clinicians and administrators can no longer afford to think of pain as an inevitable consequent of living with cancer, but rather view it as a modifiable factor that can be addressed through vigilant, evidence-based cancer screening and assessment practices.

## Trial status

Protocol Version 1.2_2018.05.29.

Recruitment to commence: 1 September 2018.

Approximate recruitment completion: 31 July 2019.

Refer to Additional file 4 for items from the World Health Organization Trial Registration Data Set.

## Additional files

Additional file 1: Participant invitation. (DOCX 87 kb)

Additional file 2: Participant Information and Consent Form (Master). (DOCX 23 kb)

Additional file 3: Qstream overview. (DOCX 489 kb)

Additional file 4: Items from the World Health Organization Trial Registration Data Set. (DOCX 15 kb)

## Abbreviations

CPD: Continuing Professional Development
HREC: Human Research Ethics Committee
IMPACCT: Improving Palliative, Aged and Chronic Care through Clinical Research and Translation
NRS: Numerical Rating Scale
NSW: New South Wales
Self-PAC: Self-Perceived Pain Assessment Capabilities
SESLHD HREC: South Eastern Sydney Local Health District Human Research Ethics Committee
UTS: University of Technology Sydney
