# Supplementary figures and images for: A phase III wait-listed randomised controlled trial of novel targeted inter-professional clinical education intervention to improve cancer patients’ reported pain outcomes (The Cancer Pain Assessment (CPAS) Trial): study protocol
Source: Trials. 2019 Jan 18;20:62. doi: 10.1186/s13063-018-3152-z (PMC6339283; doi:10.1186/s13063-018-3152-z)

**Additional file 1: Participant Invitation**


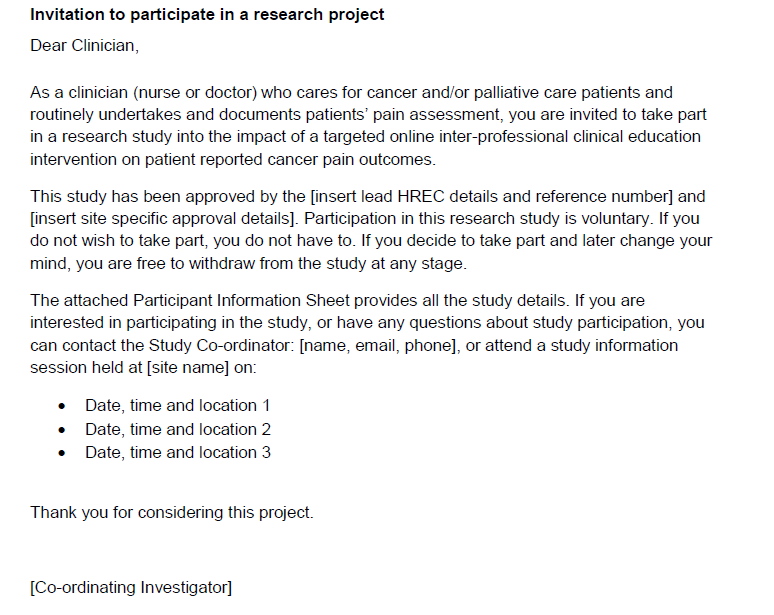

Supplement: Supplementary file 1 — Participant invitation. (DOCX 87 kb) [file 13063_2018_3152_MOESM1_ESM.docx]

**Additional file 3: Qstream Overview**


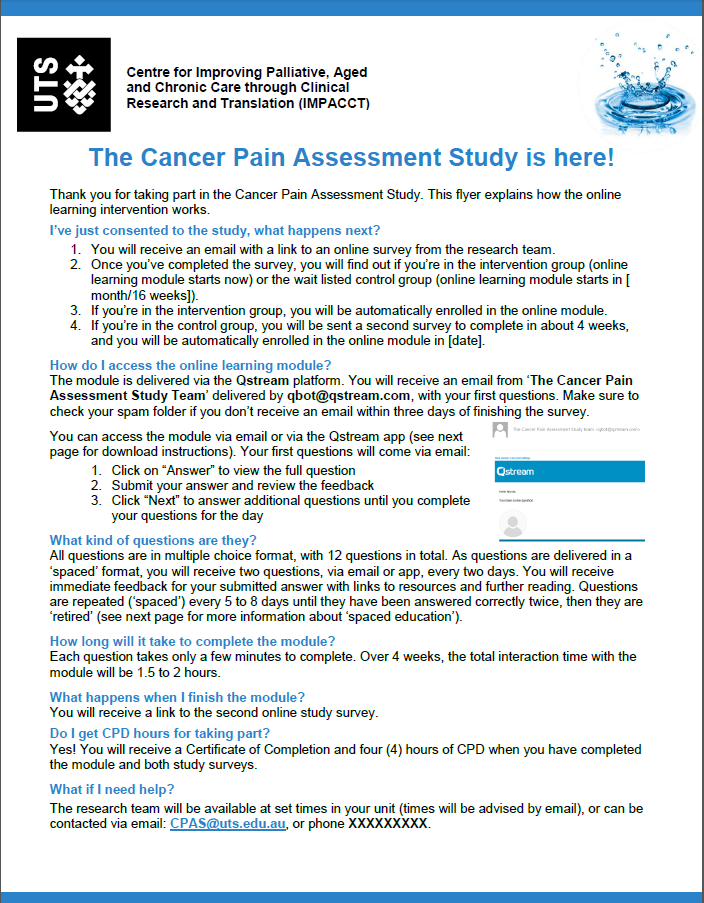

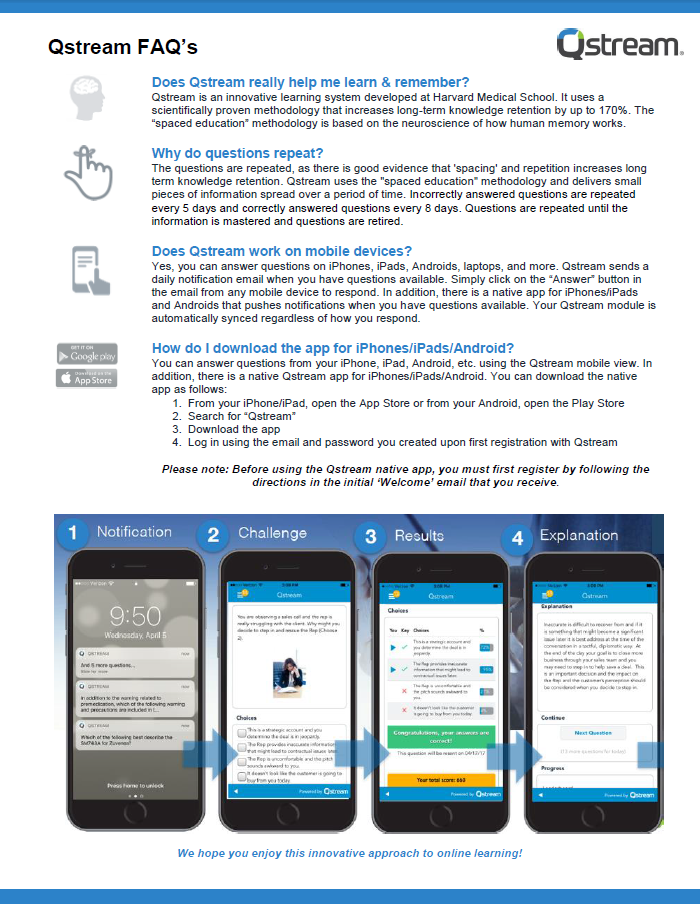

Supplement: Supplementary file 3 — Qstream overview. (DOCX 489 kb) [file 13063_2018_3152_MOESM3_ESM.docx]
