# Supplementary material for: A phase III wait-listed randomised controlled trial of novel targeted inter-professional clinical education intervention to improve cancer patients’ reported pain outcomes (The Cancer Pain Assessment (CPAS) Trial): study protocol
Source: Trials. 2019 Jan 18;20:62. doi: 10.1186/s13063-018-3152-z (PMC6339283; doi:10.1186/s13063-018-3152-z)
Supplement: Supplementary file 2 — Participant Information and Consent Form (Master). (DOCX 23 kb) [file 13063_2018_3152_MOESM2_ESM.docx]

**Additional file 2: Participant Information and Consent Form (Master)**

[Insert institutional letterhead]

[insert name of local institution/s where research is being conducted]

PARTICIPANT INFORMATION SHEET AND CONSENT FORM

CLINICAL TRIAL RESEARCH

**Improving cancer patients’ reported pain outcomes through an online inter-professional clinical education intervention.**

**Invitation**

You are invited to participate in a research study into the impact of a targeted online inter-professional clinical education intervention on patient reported cancer pain outcomes.

The study is being conducted by:

- Prof Jane Phillips, Professor Palliative Nursing, University of Technology Sydney
- Project Officer 1
- Project Officer 2

Before you decide whether or not you wish to participate in this study, it is important for you to understand why the research is being done and what it will involve. Please take the time to read the following information carefully and discuss it with others if you wish.

1. **What is the purpose of this study?**

The purpose is to investigate whether an online, clinical education module targeting clinicians pain assessment practices, can reduce cancer patients’ reported pain outcomes.

1. **Why have I been invited to participate in this study?**

You are eligible to participate in this study because you are a clinician (nurse or doctor) who cares for cancer and/or palliative care patients and routinely undertakes and documents patients’ pain assessment.

1. **What if I don’t want to take part in this study, or if I want to withdraw later?**

Participation in this study is voluntary. It is completely up to you whether or not you participate. If you decide not to participate, it will not affect your employment at your current work site.

You will be kept informed of any significant new findings that may affect your willingness to continue in the study.

If you wish to withdraw from the study once it has started, you can do so at any time without having to give a reason. However, it may not be possible to withdraw your data from the study results if these have already had your identifying details removed.

1. **What does this study involve?**

If you agree to participate in this study, you will be asked to sign the Participant Consent Form.

This study will be conducted over 16 weeks at your service.

This study is a wait listed, randomised, controlled trial. In this study, the investigators want to find out if there are any differences in clinicians’ pain assessment and documentation practices after completing the online education module. To do this, study participants are put into two groups:

- One group receives access to the online education module as soon as they consent (intervention group);
- The other group receives access to the online education module at a later date (wait listed group).

If you agree to participate in this study, you will be randomly allocated to one of these two groups. Neither the researcher nor the study participant can decide which group the participant is allocated to. If you are allocated to the intervention group, it is important that you do not discuss the questions in the online module with participants in the control group.

If you agree to participate in this trial, you will be asked to complete two online surveys:

- Survey 1: as soon as you consent (all participants)
- Survey 2: as soon as you finish the online education module (intervention group) OR four weeks after you consent (wait listed group)

You will also be required to complete the online education module. The module is made up of 10 -15 case based question with multiple choice answers. Questions are delivered via the Qstream^TM^ app (free to download to a mobile device) or directly to your nominated email account. Members of the research team will be available to assist you with downloading of the Qstream^TM^ app or enrolling in the module via email.

Once you have completed both surveys and the online education module, you will receive a certificate with four (4) hours of CPD.

1. **How is this study being paid for?**

The study is funded by a Cancer Australia Priority-driven Collaborative Cancer Research Scheme grant (Application ID: 1127011), The investigators in this study have no conflicts of interest to declare.

All of the money being paid by Cancer Australia to run the trial will be deposited into an account managed by the University of Technology Sydney. No money is paid directly to individual investigators/researchers.

1. **Are there risks to me in taking part in this study?**

The risks associated with this study are perceived to be low. The ‘Spaced Education’ on-line learning format may evoke feelings of inadequacy or discomfort associated with previous experience caring for cancer and/or palliative care patients and/or their families with complex and/or poorly managed pain. The on-line module will promote reflective practice and adoption of best evidence based pain assessment and management practices. The Research Team will be available if participants wish to explore their previous experiences and will refer participants onto appropriate support services, if required.

1. **Will I benefit from the study?**

The only direct benefit from participating in the study, other than potentially increasing your pain assessment capabilities, is receiving a certificate of completion and continuing education points, for you professional portfolio at the completion of the ‘Spaced Education’ CPD program.

1. **Will taking part in this study cost me anything, and will I be paid?**

Participation in this study will not cost you anything, and you will not be paid for participating in the study.

1. **How will my confidentiality be protected?**

Any identifiable information that is collected about you in connection with this study will remain confidential and will be disclosed only with your permission, or except as required by law. Only the project officers who are overseeing the study surveys and module enrolment will have access to your details and results. All data will be held securely at the University of Technology Sydney.

1. **What happens with the results?**

If you give us your permission by signing the consent document, we plan to discuss/publish the results in a variety of forums, including: reports to the funding body for monitoring purposes; annual reports to ethics committee(s) for monitoring purposes; peer-reviewed journals; presentation at conferences or other professional forums. In any report, publication and/or presentation, information will be provided in such a way that you cannot be identified, except with your express permission. Any publications resulting from this study will not identify your place of work. Results of the study will be provided to you, if you wish.

1. **What should I do if I want to discuss this study further before I decide?**

When you have read this information, the researcher(s) [*name(s)*] will discuss it with you and answer any queries you may have. If you would like to know more at any stage, please do not hesitate to contact the researcher(s) on [*email*].

1. **Who should I contact if I have concerns about the conduct of this study?**

This study has been approved by the South Eastern Sydney Local Health District Human Research Ethics Committee. Any person with concerns or complaints about the conduct of this study should contact the Research Support Office which is nominated to receive complaints from research participants. You should contact them on 02 9382 3587, or email [SESLHD-RSO@health.nsw.gov.au](mailto:SESLHD-RSO@health.nsw.gov.au) and quote 17/322 (HREC project number).

The conduct of this study at the [*name of site*] has been authorised by the [*name of health district*]. Any person with concerns or complaints about the conduct of this study may also contact the [*details of the Research Governance Officer of the health district*]

**Thank you for taking the time to consider this study.**

**If you wish to take part in it, please sign the attached consent form.**

**This information sheet is for you to keep.**

***[Insert institutional letterhead]***

***[name of local institution/s where research is being conducted]***

**CONSENT FORM**

[To be used in conjunction with a Participant Information Sheet]

**Improving cancer patients’ reported pain outcomes through an online inter-professional clinical education intervention.**

1. I,................................................................................................................. of................................................................................................................

agree to participate in the study described in the participant information statement set out above***.***

2. I acknowledge that I have read the participant information statement, which explains why I have been selected, the aims of the study and the nature and the possible risks of the investigation, and the statement has been explained to me to my satisfaction.

3. Before signing this consent form, I have been given the opportunity of asking any questions relating to any possible physical and mental harm I might suffer as a result of my participation and I have received satisfactory answers.

4. I understand that I can withdraw from the study at any time without prejudice to my relationship to [hospital site].

5. I agree that research data gathered from the results of the study may be published, provided that I cannot be identified.

6. I understand that if I have any questions relating to my participation in this research, I may contact [Project Officer] on telephone................., who will be happy to answer them.

1. I acknowledge receipt of a copy of this Consent Form and the Participant Information Statement.

Complaints may be directed to the Research Ethics Secretariat, South Eastern Sydney Local Health District, Prince of Wales Hospital, Randwick NSW 2031 Australia (phone 02-9382 3587, fax 02-9382 2813, email [SESLHD-RSO@health.nsw.gov.au](mailto:SESLHD-RSO@health.nsw.gov.au) .

Signature of participant Please PRINT name Date

_________________________ _______________________ _______________

Signature of witness Please PRINT name Date

_________________________ _______________________ _______________

Signature of investigator Please PRINT name Date

_________________________ _______________________ _______________

[Institutional letterhead]

[Insert name of local institution where research is being conducted]

**Improving cancer patients’ reported pain outcomes through an online inter-professional clinical education intervention.**

**WITHDRAWAL OF CONSENT**

**I hereby wish to WITHDRAW my consent to participate in the study described above and understand that such withdrawal WILL NOT jeopardise any treatment or my relationship with the [site name].**

**Signature of participant Please PRINT name Date**

**_________________________ _______________________ _______________**

The section for Revocation of Consent should be forwarded to:

Prof Jane Phillips

Director IMPACCT

Faculty of Health

University of Technology, Sydney

PO Box 123

Broadway NSW 2007 Australia
