# Supplementary material for: A phase III wait-listed randomised controlled trial of novel targeted inter-professional clinical education intervention to improve cancer patients’ reported pain outcomes (The Cancer Pain Assessment (CPAS) Trial): study protocol
Source: Trials. 2019 Jan 18;20:62. doi: 10.1186/s13063-018-3152-z (PMC6339283; doi:10.1186/s13063-018-3152-z)
Supplement: Supplementary file 4 — Items from the World Health Organization Trial Registration Data Set. (DOCX 15 kb) [file 13063_2018_3152_MOESM4_ESM.docx]

**Additional file 4: Items from the World Health Organization Trial Registration Data Set**

| **Data category** | **Information** |
| --- | --- |
| Primary registry and trial identifying number | Australian New Zealand Clinical Trials Registry (ANZCTR): ACTRN12618001103257 |
| Date of registration in primary registry | July 3, 2018 |
| Secondary identifying numbers | n/a |
| Source(s) of monetary or material support | Cancer Australia: Priority-driven Collaborative Cancer Research Scheme; Grant ID: #1127011 |
| Primary sponsor | University of Technology Sydney |
| Secondary sponsor(s) | Nil |
| Contact for public queries | CPAS@uts.edu.au |
| Contact for scientific queries | CPAS@uts.edu.au |
| Public title | Improving cancer patients' reported pain outcomes through clinician mHealth training - a randomised controlled trial. |
| Scientific title | A phase III wait-listed RCT of a novel targeted inter-professional clinical education intervention to improve cancer patients’ reported pain outcomes. |
| Countries of recruitment | Australia |
| Health condition(s) or problem(s) studied | Cancer pain |
| Intervention(s) | Clinician-focused, spaced learning pain assessment performance feedback intervention delivered via the Qstream™ platform |
| Key inclusion and exclusion criteria | Ages eligible for study: ≥18 years  Sexes eligible for study: both Accepts healthy volunteers: yes |
|  | Inclusion criteria: All medical and nursing personnel routinely caring for cancer and/or palliative care patients at a participating site are eligible to participate in the study. Participants must be willing to give written informed consent, and willing to participate to and comply with the study. |
|  | Exclusion criteria: Agency staff; casual staff who have worked less than one shift in the month before the intervention commences; Unregistered health professional who are unlikely to be undertaking and documenting patients’ pain assessment, e.g., Aged Care Workers (ACW), Personal Care Assistants (PCA), Care Support Employees (CSE) and Health Services Assistants (HSA). |
| Study type | Wait listed, randomised controlled trial |
|  | Allocation: Simple randomisation using a randomisation table created by computer software |
|  | Primary purpose: Educational / counselling / training |
|  | Phase III |
| Date of first enrolment | Proposed September 1, 2018 |
| Target sample size | 90 |
| Recruitment status | Recruitment commencing September 2018. |
| Primary outcome(s) | Mean change in patients’ pain numerical rating score ("NRS') (0–10) scores. |
| Key secondary outcomes | Clinicians' pain screening/assessment adherence score; comprehensive pain assessment quality documentation score; Self-Perceived Pain Assessment Capabilities (Self-PAC); economic evaluation of intervention |
